# Positioning Filgotinib in the Treatment Algorithm of Moderate to Severe Ulcerative Colitis

**DOI:** 10.1093/ecco-jcc/jjab206

**Published:** 2021-11-16

**Authors:** Ferdinando D’Amico, Fernando Magro, Laurent Peyrin-Biroulet, Silvio Danese

**Affiliations:** Department of Biomedical Sciences, Humanitas University, Pieve Emanuele, Milan, Italy; Gastroenterology and Endoscopy, IRCCS Ospedale San Raffaele and Vita-Salute San Raffaele University, Milan, Italy; Department of Gastroenterology, Centro Hospitalar São João, Porto, Portugal; Department of Gastroenterology and Inserm NGERE U1256, University Hospital of Nancy, University of Lorraine, Vandoeuvre-lès-Nancy, France; Gastroenterology and Endoscopy, IRCCS Ospedale San Raffaele and Vita-Salute San Raffaele University, Milan, Italy

**Keywords:** Filgotinib, small molecule, JAK inhibitor, ulcerative colitis, inflammatory bowel disease

## Abstract

**Background and Aims:**

Filgotinib is a small molecule that selectively inhibits Janus kinase [JAK] type 1. It is already approved for the treatment of rheumatoid arthritis and is being evaluated for the management of patients with moderate to severe ulcerative colitis [UC]. The purpose of this review is to provide an overview of the currently available data on filgotinib and to define how to position this new drug in the treatment algorithm of patients with UC.

**Methods:**

The Pubmed, Embase and Scopus databases were searched up to June 25, 2021 in order to identify studies reporting efficacy and safety data of filgotinib in patients with UC.

**Results:**

Data from a phase III study enrolling UC patients with moderate to severe disease show that filgotinib is effective with a reassuring safety profile. Filgotinib treatment is not associated with a greater risk of thrombosis and herpes zoster infections compared to other JAK inhibitors. However, animal studies reported impaired spermatogenesis and histopathological effects on male reproductive organs, making it necessary to deepen this aspect in dedicated human studies.

**Conclusions:**

Filgotinib is an effective and safe drug for treatment of both biologic-naive and biologic-experienced patients with moderate to severe UC and may soon be available.

## 1. Introduction

Ulcerative colitis [UC] is a chronic inflammatory bowel disease [IBD] with a remitting and relapsing course.^1^ In the last few decades, the therapeutic options for patients with UC have substantially increased with the introduction of biological therapies. Several drugs are currently available including tumour necrosis factor [TNF] inhibitors [golimumab, adalimumab and infliximab], integrin inhibitors [vedolizumab] and inhibitors of interleukin 12–23 [ustekinumab].^2^ However, up to 30% of patients fail to respond to initial therapy and roughly 50% lose response over time, with 10% still requiring surgery.^3^ Several efforts have been made to develop new molecules and to address this unmet medical need.^4^ In 2018, tofacitinib, a small molecule, was approved by the Food and Drug Administration [FDA] and the European Medicines Agency [EMA] for the treatment of UC.^5^ Tofacitinib is an oral drug and has a completely new mechanism of action.^6^ Unlike biologics that specifically block a certain target, tofacitinib interferes with the janus kinase [JAK]/signal transducer and activator of transcription [STAT] pathways. JAKs comprise four intracellular tyrosine kinases [JAK1, JAK2, JAK3 and TYK2] which regulate different intracellular functions including inflammatory mechanisms.^7,8^ They stimulate the activity of lymphocytes and cytokines and the production of mucus and have a relevant role in haematopoiesis and viral defence.^7^ The inhibition of JAK allows us to modulate different components of the inflammation at the same time as contributing to a reduction of the inflammatory state.^7,8^ Tofacitinib inhibits JAK1 and JAK3 but at high concentrations it also blocks JAK2 and TYK2 and can be considered a pan-JAK inhibitor.^7^ Phase III clinical trials have proved the efficacy of tofacitinib in patients with moderate to severe UC.^9–12^ However, concerns about the safety profile persist with regard to a reported increased risk of high lipid concentrations, thromboembolic events and herpes zoster infections.^13–15^ Interestingly, growing evidence shows that JAK1 is the pathway most implicated in innate and adaptive immune responses.^16^ On the other hand, JAK2 is related to erythropoiesis and thrombopoiesis, JAK3 to lymphocyte proliferation and immune homeostasis, while TYK2 is associated with antiviral responses.^16^ It is therefore plausible that the selective inhibition of JAK1 may be associated with an improved safety profile and a reduced rate of adverse events. This rationale led to the development of filgotinib, a selective inhibitor of JAK1. Filgotinib proved to be effective for the treatment of rheumatoid arthritis and is approved for this indication.^17^ The efficacy and safety of filgotinib has also been tested in the field of IBD.^18,19^ The purpose of this review is to provide an overview of the available evidence on filgotinib in patients with moderate to severe UC in order to better define how to position this new small molecule into the therapeutic algorithm of UC.

## 2. Review criteria

We searched the Pubmed, Embase and Scopus databases up to June 25, 2021 in order to identify studies reporting efficacy and safety data of filgotinib in patients with UC. The following search terms were used: ‘filgotinib’, ‘JAK inhibitors’, ‘anti-JAK’, ‘selective JAKi’ combined with ‘ulcerative colitis’, ‘UC’, ‘inflammatory bowel disease’ and ‘IBD’. Only articles published in English were considered. Three authors [F.D., S.D. and L.P.B.] independently reviewed titles and abstracts to identify eligible studies. The full texts of the selected articles were examined for inclusion, and relevant references in their lists were hand searched to identify studies missed by the electronic search. Abstracts and articles were included based on their relevance.

## 3. Pharmacokinetics and pharmacodynamics

Filgotinib has a 5-fold higher potency of inhibiting JAK1 compared to JAK2, JAK3 and TYK2 and can be considered a selective JAK1 blocker^17^ [Table 1]. Filgotinib is rapidly absorbed and its median peak plasma concentration [*C*_max_] is reached 2–3 h after the drug dose.^17^ Its mean half-life is approximately 7 h.^17^ No difference in filgotinib concentrations was found based on drug administration in combination with high or low fat or fasting meals.^17^ Thus, food does not affect filgotinib concentrations and the drug can be given with or without food.^17^ Filgotinib binding to human plasma proteins is low [55–59%] suggesting that this small molecule has no preferential distribution within blood cells.^17^ Filgotinib is predominantly metabolized by carboxylesterase 2 [CES2] and CES1, in a non-CYP450-dependent fashion, leading to the production of its active metabolite, GS-829845, with similar JAK1 selectivity, which is approximately 10-fold less potent, but a 16-20-fold higher exposure than the parent molecule.^17^ Elimination of the drug is mainly urinary [85%], while a small part is eliminated in the faeces [15%].^17^ No clinically significant differences were detected in patients treated with filgotinib or its metabolite based on bodyweight, gender, race or age.^17^ Furthermore, the pharmacokinetics of filgotinib was not changed in patients with mild renal disease (creatinine clearance [CrCl] between 60 and <90 mL/min] or with moderate liver disease [Child-Pugh B], while in subjects with moderate [CrCl 30 to <60 mL/min] or severe [CrCl 15 to <30 mL/min] renal disease an increased drug concentration was detected [≤ 2-fold and 2.2-fold increase, respectively], and therefore a lower dose [100 mg] is recommended for this group of patients.^17^ The effects of the drug on patients with end-stage renal disease [CrCl <15 mL/min] and severe hepatic impairment [Child-Pugh C] have not been studied, while both filgotinib and its metabolite have not been associated with prolongation of the corrected QT interval.^17,20^ Regarding the interactions between filgotinib and other molecules, it should be emphasized that filgotinib does not significantly inhibit or induce cytochrome P450 [CYP] enzymes or UDP-glucuronosyltransferases, which are generally involved in drug interactions, suggesting a low risk of interaction with other drugs.^21–24^

**Table 1.** Pharmacokinetic and pharmacodynamic characteristics of filgotinib

Administration route	Oral
JAK selectivity	JAK 1
Median peak plasma concentration	2–3 h
Half-life	7 h
Correlation with food	Food does not affect filgotinib concentrations
Binding to human plasma proteins	55–59%
Metabolization	Carboxylesterase 2 and carboxylesterase 1
Metabolite	GS-829845
Urinary elimination Faecal elimination	85% 15%
Renal impairment • CrCl between 60 and <90 mL/min • CrCl 30 to <60 mL/min • CrCl 15 to <30 mL/min • CrCl < 15 mL/min	No dose adjustment Adjustment to filgotinib 100 mg per day Adjustment to filgotinib 100 mg per day Not known
Liver disease • Child-Pugh A • Child-Pugh B • Child-Pugh C	No dose adjustment No dose adjustment Not known
Bodyweight Gender Race	No dose adjustment No dose adjustment No dose adjustment
Immunogenicity	None

CrCl: creatinine clearance.

## 4. Efficacy and safety in UC

The efficacy and safety of filgotinib were tested in a randomized, double-blind phase 2b/3 study [SELECTION trial] enrolling patients with moderately to severely active UC.^25–27^ The trial consisted of two induction studies and one maintenance study. In the induction study, both biologically naive patients and those who had previously failed biologic therapy were eligible. In total, 659 biologic-naïve and 689 biologic-experienced patients [of whom 43.1% had experienced failure of both a TNF antagonist and vedolizumab] were included and randomized 2:2:1 into three groups [filgotinib 200 mg daily, filgotinib 100 mg daily or placebo].^27^ The primary endpoint was clinical remission at week 10, defined as Mayo endoscopic subscore ≤ 1, rectal bleeding subscore = 0, and ≥ 1-point decrease in stool frequency subscore from baseline to achieve a subscore ≤ 1. Clinical remission at week 10 was achieved in a significantly higher proportion of biologic-naïve [26.1% vs 15.3%, *p* = 0.0157] and biologic-experienced [11.5% vs 4.2%, *p* = 0.0103] subjects treated with filgotinib 200 mg compared with placebo. Additionally, a higher rate of Mayo clinic score remission [defined as a total MCS ≤2 with no single subscore >1], endoscopic remission [Mayo endoscopic subscore = 0] and histological remission [no neutrophils in the lamina propria or epithelium, no crypt destruction, erosion, ulceration or granulation tissue using Geboes Index] at week 10 were detected in biologic-naïve [24.5% vs 12.4%, *p* = 0.0053; 12.2% vs 3.6%, *p* = 0.0047; 35.1% vs 16.1%, *p* < 0.0001] and biologic-experienced [9.5% vs 4.2%, *p* > 0.05; 3.4% vs 2.1%, *p* > 0.05; 19.8% vs 8.5%, *p* > 0.05] patients treated with filgotinib 200 mg compared with placebo. Interestingly, the rate of adverse events was similar between the filgotinib 200 and 100 mg groups and placebo arm [53.6% and 50.4% vs 56.3%]. Similarly, no difference in the rate of serious adverse events [4.3% and 5.0% vs 4.7%] and serious infections [0.6% and 1.1% vs 1.1%] was found. Herpes zoster infections occurred in four patients (one in the filgotinib 100 mg group [0.2%] and three in the filgotinib 200 mg group [0.6%]), while only one case of pulmonary embolism was detected in the filgotinib 200 mg arm [0.2%]. Patients who achieved clinical remission or clinical response after 10 weeks were included in the maintenance study. Subjects treated with filgotinib during the induction phase were rerandomized 2:1 to induction filgotinib dose or placebo, while patients previously randomized to placebo continued placebo. The primary endpoint was clinical remission at week 58 defined as Mayo endoscopic subscore ≤ 1, rectal bleeding subscore = 0 and ≥ 1-point decrease in stool frequency from induction baseline to achieve a subscore ≤ 1. Finally, 664 patients were included in the maintenance study. Clinical remission occurred in a significantly higher proportion in the filgotinib 200 and 100 mg arms than in the placebo groups [37.2% and 23.8% vs 11.2% and 13.5%, *p* < 0.025 for both comparisons]. Filgotinib 200 mg was associated with a significant higher rate of endoscopic [15.6% vs 6.1%, *p* < 0.025] and histological [38.2% vs 13.3%, *p* < 0.025] remission compared with placebo. Similarly, a numerically higher proportion of subjects in the filgotinib 100 mg group achieved endoscopic [13.4% vs 7.9%, *p* > 0.05] and histological [27.9% vs 18.0%, *p* > 0.05] remission compared with placebo. Interestingly, a higher percentage of Mayo clinic score remission was found in the filgotinib 200 mg group compared to placebo as early as week 4 in both biologic-naive [30.6% vs 16.8%, *p* = 0.0035] and biologic-experienced patients [21.0% vs 7.7%, *p* = 0.0005].^28^ This improvement remained stable over time throughout the maintenance phase.^28^ Moreover, greater improvements from baseline in quality of life based on the 36-item short form survey 36 [SF-36] score were experienced in both biologic-naive and biologic-exposed patients treated with the experimental drug compared with placebo both after 10 weeks of induction therapy and after 58 weeks of maintenance therapy.^29^ No significant difference in the incidence of adverse events [60.3% and 66.8% vs 61.3%, *p* > 0.05] and serious adverse events [4.5% and 4.5% vs 4.3%, *p* > 0.05] was found. No venous thromboses or pulmonary embolisms were diagnosed in filgotinib-treated patients and the incidence of herpes zoster was very low [<1%]. Importantly, two subjects on filgotinib 200 mg died, but the causes were unrelated to the experimental drug according to the investigators’ opinion. Data from the filgotinib UC programme were recently reported including 1069 patients exposed to filgotinib in induction, maintenance and long-term extension [LTE] studies and 279 subjects treated with placebo in the induction phase.^30^ Similar rates of herpes zoster infections [0.3 exposure-adjusted event rates per 100 patient-years vs 0.3 and 1.8], venous thrombosis [0.9 vs 0.0 and 0.2] and serious infections [2.2 vs 3.5 and 2.2] occurred among patients receiving placebo, filgotinib 100 mg or filgotinib 200 mg, highlighting its adequate safety profile.

## 5. Safety in other immune-mediated inflammatory disorders

Most of the literature evidence on filgotinib comes from rheumatological studies. In the EQUATOR trial, a randomized, double-blind, placebo-controlled phase 2 trial enrolling adult patients with active moderate-to-severe psoriatic arthritis, no significant difference in the rate of treatment-emergent adverse events [57% vs 59%] and serious adverse events [2% vs 2%] was detected between filgotinib and placebo during the study period.^31^ Nasopharyngitis and headache were the most frequent adverse events. Similarly, the same proportion of adverse events were found in patients with ankylosing spondylitis after being randomized to take filgotinib or placebo [31% vs 31%].^32^ Nasopharyngitis was the most frequent adverse event, while no cases of herpes zoster infection or neoplasia were reported. One case of non-serious deep vein thrombosis occurred in a 53-year-old patient with a factor V Leiden mutation receiving filgotinib therapy. Several studies investigated the safety of filgotinib in patients with rheumatoid arthritis.^31,33–36^ DARWIN I was a 24-week phase IIb randomized clinical trial that evaluated the efficacy and safety of different doses of filgotinib [once or twice-daily 50, 100 or 200 mg] as add-on treatment to methotrexate in 594 patients with active rheumatoid arthritis.^33^ At the end of the 24-week study, similar proportions of adverse events were found between patients in the placebo group or once-twice-daily filgotinib 50, 100 or 200 mg groups [57.1% vs 52.4%, 43.5% and 58.1% or 53.6%, 54.1% and 53.6%]. Eleven subjects treated with filgotinib experienced serious adverse events compared with four in the placebo group [1.3% vs 7.1%] including one potentially drug-related death from pneumonia and septic shock, two major cardiovascular events, and five herpes zoster infections. The DARWIN II trial assessed the efficacy and safety of filgotinib monotherapy in 283 patients with rheumatoid arthritis.^34^ No significant difference in the rate of adverse events was found between placebo and filgotinib 50, 100 or 200 mg at week 12 [38.9% vs 40.3%, 32.9% and 43.5% respectively]. Severe adverse events occurred in eight patients in the experimental group at the end of the follow up (two filgotinib 50 mg-treated patients [3.5%], two filgotinib 100 mg-treated patients [2.9%] and three filgotinib 200 mg-treated patients [4.3%]). No deaths occurred after 24 weeks. Additionally, a case of herpes zoster infection was recorded in a patient treated with JAK inhibitor. Data from phase III studies on filgotinib in rheumatoid arthritis [FINCH I, II and III] revealed that nasopharyngitis was the most frequent adverse event. In the FINCH I trial, patients were randomized to filgotinib 100 mg, filgotinib 200 mg, active comparator [adalimumab] or placebo.^36^ There was no difference in the rate of serious adverse events between the study arms [4.2% in the placebo arm, 4.3% in the adalimumab arm, and 4.4% and 5.0% in the filgotinib 200 mg and 100 mg arms respectively]. Herpes zoster infections were diagnosed in four patients treated with filgotinib [two in the 100 mg arm and 2 in the 200 mg arm], while one case of major cardiovascular events and neoplasia was found in the low-dose filgotinib group and one case of deep vein thrombosis in the higher dose group. In FINCH II, subjects were exposed to filgotinib 100 or 200 mg or placebo.^37^ Four cases of uncomplicated herpes zoster, one retinal vein occlusion and two major cardiovascular events were detected. Finally, in FINCH III, patients were randomized into four groups: filgotinib 100 mg + methotrexate, filgotinib 200 mg + methotrexate, filgotinib 200 mg alone and methotrexate alone.^35^ Adverse events of particular interest in the filgotinib monotherapy group were one case of herpes zoster infection and one major cardiovascular event, while neither thrombotic events nor tumours or deaths were reported. Importantly, safety analyses from an open-label extension study of phase 2 rheumatoid arthritis programmes showed that exposure-adjusted incidence rates of treatment-emergent adverse events [TEAEs] and serious TEAEs per 100 patient years of exposure were 24.6 and 3.1 in the filgotinib + methotrexate group and 25.8 and 4.3 in the filgotinib monotherapy group, respectively.^38^ The FITZROY was a randomized, double-blind, placebo-controlled phase 2 trial that investigated the efficacy and safety of filgotinib in 174 patients with moderate-to-severe Crohn’s disease [Table 2].^18^ The study consisted of two phases. Initially, patients were randomized [3:1] to filgotinib 200 mg or placebo for 10 weeks. Then, patients were divided into three arms based on the clinical response: filgotinib 200 mg, filgotinib 100 mg or placebo for an additional 10 weeks. In the pooled analysis, the proportion of TEAEs and serious TEAEs was similar between the filgotinib and placebo groups [75% and 9% vs 67% and 4% respectively], including an increase in levels of high-density lipoprotein [HDL] cholesterol and low-density lipoprotein [LDL] cholesterol [11% and 12% vs 4% and 13%]. Interestingly, a numerically higher proportion of serious infections was reported in the pooled filgotinib group than in placebo-treated patients [3% vs 0%], while only one case of herpes zoster infection was found in the filgotinib arm. Importantly, animal studies reported that some male subjects treated with filgotinib had minimal to moderate testicular atrophy/degeneration in the epididymis.^17^ The FDA recently requested data to investigate whether filgotinib has an impact on sperm parameters.^39^ A randomized phase II trial in UC patients [NCT03201445] is currently evaluating the testicular safety profile of filgotinib. Another study [MANTA-Ray] recently provided preliminary data about male reproductive safety in filgotinib-treated patients with active rheumatoid arthritis, psoriatic arthritis, ankylosing spondylitis and non-radiographic axial spondyloarthritis. Of note, 8.3% of patients on placebo and 6.7% of patients on filgotinib had a 50% or more decline in sperm concentration at week 13 and no new safety findings were identified.^40^

**Table 2.** Safety of filgotinib in patients with immune-mediated inflammatory disorders

First author	Study population	Study design	Study duration	AEs	SAEs	Increase in HDL	Increase in LDL	HZ infection	DVT	PE	MACEs	Malignancies
Vermeire^18^	174 CD	RCT	20 weeks	75% FIL 67% PBO	9% FIL 4% PBO	11% FIL 4% PBO	12% FIL 13% PBO	0.7% FIL 0% PBO	NA	NA	NA	NA
Mease^31^	131 PsoA	RCT	16 weeks	57% FIL 59% PBO	2% FIL 2% PBO	NA	NA	2% FIL 0% PBO	0% FIL 0% PBO	0% FIL 0% PBO	2% FIL 0% PBO	0% FIL 0% PBO
van der Heijde^32^	116 AS	RCT	12 weeks	31% FIL 31% PBO	3% FIL 0% PBO	NA	NA	0% FIL 0% PBO	2% FIL 0% PBO	0% FIL 0% PBO	0% FIL 0% PBO	0% FIL 0% PBO
Combe^36^	1755 RA	RCT	24 weeks	NA	4.4% FIL 4.2% PBO	NA	NA	0.4% FIL 0.4% PBO	0.2% FIL 0.4% PBO	0% FIL 0% PBO	0% FIL 0.4% PBO	0% FIL 0.6% PBO
Genovese^a^^37^	449 RA	RCT	24 weeks	69% FIL 68% PBO	4.1% FIL 3.4% PBO	NA	NA	1.4% FIL 0% PBO	0.7% FIL 0% PBO	0% FIL 0% PBO	0% FIL 0.7% PBO	0% FIL 0% PBO
Westhovens^a35^	1249 RA	RCT	24 weeks	NA	4.8% FIL	NA	NA	0.5%	0%	0%	0%	0%
Westhovens^a33^	594 RA	RCT	24 weeks	58% FIL 57% PBO	2.3% FIL 7.1% PBO	NA	7% FIL 9% PBO	0.1% FIL 0.2% PBO	0% FIL 0% PBO	0% FIL 0% PBO	0% FIL 0% PBO	0% FIL 0% PBO
Kavanaugh^a34^	283 RA	RCT	24 weeks	50.7% FIL 39% PBO	4.1% FIL 1.4% PBO	NA	8.7% FIL 5.6% PBO	0% FIL 0% PBO	0% FIL 0% PBO	0% FIL 0% PBO	0% FIL 0% PBO	0% FIL 0% PBO

CD: Crohn’s disease; AS: ankylosing spondylitis; RA: rheumatoid arthritis; AEs: adverse events: SAEs: serious adverse events; PsoA: psoriatic arthritis; HZ: herpes zoster; RCT: randomized controlled trial; FIL: filgotinib; PBO: placebo; HDL: high-density lipoprotein; LDL: low-density lipoprotein; NA: not available; MACEs: major adverse cardiovascular events; PE: pulmonary embolism; DVT: deep venous thrombosis.

^a^Only safety data on patients treated with filgotinib 200 mg are reported.

## 6. Positioning filgotinib in UC

**Figure 1.**
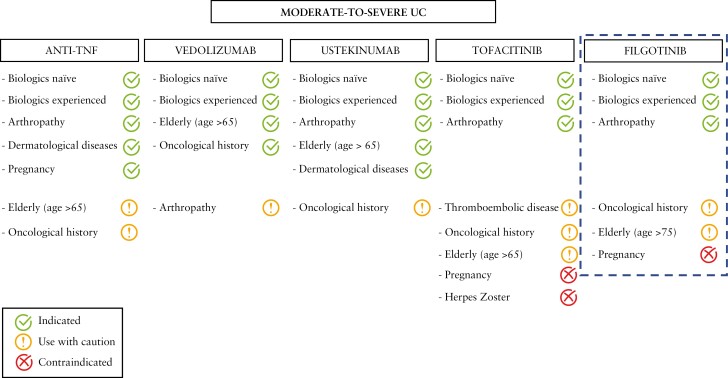
Proposed algorithm for a tailored therapy with biologic agents or small molecules in moderate-to-severe UC. UC: ulcerative colitis; TNF: tumour necrosis factor. Dashed line: currently under investigation.

**Figure 2.**
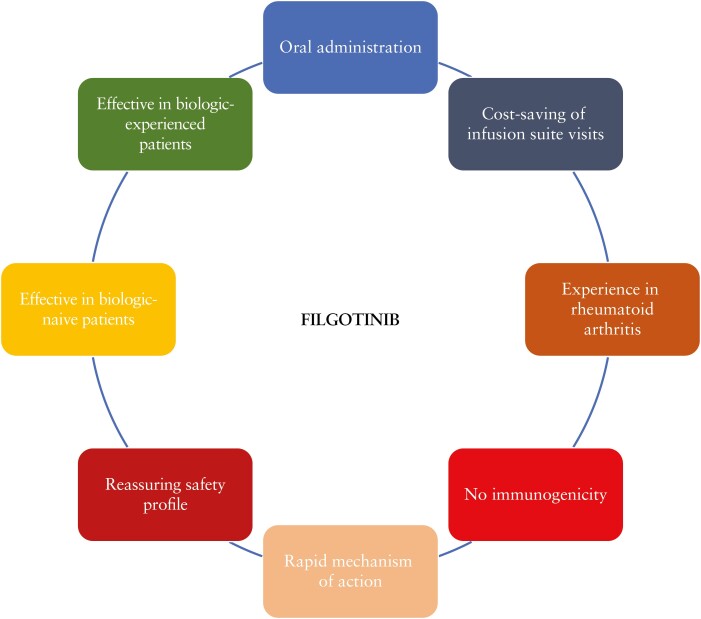
Advantages of filgotinib use in patients with moderate to severe ulcerative colitis.

In recent decades, the leitmotif of pharmacological research has been the need for effective but at the same time safe drugs. The development of JAK inhibitors represents a revolution in the treatment of patients with UC. Filgotinib is an innovative drug and could represent a valid alternative to currently available molecules. It has a rapid mechanism of action and oral administration is generally well accepted by patients.^17^ However, it remains to be determined where to position this drug in the therapeutic algorithm of UC patients and what advantage filgotinib can offer over currently approved drugs. To date, a specific and validated algorithm for the management of patients with moderate to severe UC is not available. It is important to underline that the positioning of filgotinib in rheumatology is much clearer. In fact, in rheumatoid arthritis, JAK inhibitors are commonly used after failure of conventional disease-modifying antirheumatic drugs [DMARDs] [e.g. methotrexate, leflunomide, sulfasalazine and hydroxychloroquine].^41^ JAK inhibitors can be added to conventional therapy in case of poor prognostic factors or be used to replace traditional therapy if there are no risk factors.^41^ In patients with UC, on the other hand, there is little evidence on this topic. This can be explained by the lack of head-to-head trials that provide a direct comparison between drugs. In the absence of a commonly accepted therapeutic strategy, it is likely that therapeutic decisions will be increasingly individualized and tailored according to the patient’s characteristics [Figure 1]. In elderly subjects or those with a previous history of malignancy, the use of vedolizumab, a drug with a selective gut mechanism, may be preferred for its safety profile. Conversely, the benefit of anti-integrin in those with extraintestinal manifestations [e.g. joint symptoms] is unclear.^42^ In this context, TNF alpha inhibitors, ustekinumab or JAK inhibitors may be indicated. JAK inhibitors are not recommended during pregnancy as they are associated with a risk of teratogenicity.^43^ On the other hand, TNF blockers are commonly used during pregnancy and the first efficacy and safety data of vedolizumab and ustekinumab in pregnant women have been reported.^44,45^ The rapidity of action of filgotinib could justify its choice given that as early as from 4 weeks of treatment it is associated with significant clinical improvements leading to a better quality of life. The lack of immunogenicity is another point in favour of filgotinib, as unlike biological drugs its efficacy is not affected by drug concentration and the development of anti-drug antibodies. Of note, patient preference is another variable to consider in the therapeutic decision-making process. Most UC patients are young of working age so infusion medications may be challenging and limit their quality of life.^46^ Some patients are afraid of needles, hindering the use of subcutaneous drugs.^47^ The use of oral drugs could reduce the indirect treatment costs as it could reduce hospital admissions and overcrowding by preventing the risks of viral infection in the current global pandemic context. Finally, filgotinib could be used in both biologic-naive and biologic-exposed patients. It could represent a valid alternative to tofacitinib, particularly in those who have an increased thromboembolic or infectious risk [Figure 2]. Filgotinib could be used in steroid-resistant and steroid-dependent patients or those unresponsive to immunosuppressive drugs. To date, comparative data between immunosuppressants and small molecules are not available but anti-JAK agents may be preferred due to their ability to induce disease remission and the questionable handling of immunosuppressants, which require periodic biochemical tests and have been associated with an increased risk of haematological disorders and malignancies.^48–50^

## 7. Filgotinib vs other JAK inhibitors

Most of the data comparing JAK inhibitors come from rheumatoid arthritis studies. A network meta-analysis including five randomized clinical trials with over 1500 patients with rheumatoid arthritis compared the efficacy and safety data of tofacitinib, baricitinib, upadacitinib, filgotinib and peficitinib as monotherapy.^51^ All the small molecules proved to be effective.^51^ Interestingly, peficitinib had the highest probability of being the most effective drug, followed by filgotinib and then by tofacitinib, upadacitinib and baricitinib. No significant differences in the adverse event rate were recorded.^51^ Sung and Lee investigated the efficacy and safety of tofacitinib, baricitinib, upadacitinib and filgotinib in disease-modifying antirheumatic drug-naive patients with rheumatoid arthritis.^52^ Patients treated with tofacitinib 5 mg had the highest probability of achieving the American College of Rheumatology 50 and American College of Rheumatology 70 response rates, while adverse events occurred in a similar percentage among the study arms. Another network meta-analysis of four randomized trials comprising more than 5000 patients with rheumatoid arthritis with inadequate responses to methotrexate evaluated the efficacy and safety of tofacitinib, baricitinib, upadacitinib, filgotinib and adalimumab.^53^ Baricitinib and upadacitinib showed the best efficacy data based on the American College of Rheumatology 20% [ACR20] response rate, while filgotinib was associated with the lowest probability of developing herpes zoster infections according to the surface under the cumulative ranking curve [SUCRA].^53^ Similarly, a meta-analysis by Lee and colleagues reported that tofacitinib 5 mg and filgotinib 200 mg were associated with a significantly lower serious adverse event rate than upadacitinib 15 mg in subjects refractory to biologic disease-modifying antirheumatic drugs (odds ratios [OD] 0.02, 95% confidence interval [CI] 0.00–0.30, and OD 0.08, 95% CI 0.00–0.93, respectively).^54^ A recent meta-analysis including patients with immune-mediated inflammatory disorders revealed that JAK inhibitors were associated with an increased risk of herpes zoster infection.^55^ In particular, tofacitinib [a JAK1 and JAK3 inhibitor] and baricitinib [a JAK1 and JAK2 inhibitor] led to a greater risk of herpes zoster infections (relative risk [RR] 1.50, CI 95% 0.76–2.96, and RR 2.05, 95% CI 0.99–4.24] compared to filgotinib [RR 1.28, 95% CI 0.32–5.07] suggesting that the selectivity of filgotinib for JAK1 may account for the reduced risk of infections. In addition, most diagnosed herpes zoster infections were mild to moderate in severity. However, it must be emphasized that further studies are needed to confirm the link between drug selectivity and safety. Furthermore, the drug selectivity is lost with the dose increase.^7^ To overcome this limitation, patients who are candidates for JAK inhibitor therapy should be vaccinated for herpes zoster before starting therapy in order to minimize the risk of infection.^56,57^ Finally, an increased risk of thromboembolic events has been recorded in rheumatoid arthritis patients with cardiovascular risk factors who are undergoing tofacitinib therapy.^58^ Following this finding, the regulatory activities of the FDA launched a warning stressing the need to evaluate the risks and benefits of tofacitinib in patients with high thrombotic risk and imposing the use of the lowest drug dosage available in this setting.^58^ As noted above, few cases of pulmonary thromboembolism have also been reported in patients treated with filgotinib, but whether this is a drug class adverse event or due to inhibition of a specific pathway is not yet known and needs to be confirmed in the long term.

## 8. Discussion

Treatment scenarios for UC patients are rapidly expanding. The need for safer drugs has led to the development of selective drugs in order to reduce the occurrence of side effects. In addition, increasing attention is given to the rapidity of action of the drug and to the administration route, aiming at providing a benefit in the shortest time and improving the quality of life of patients. Filgotinib is a second-generation JAK inhibitor, which is administered orally and has a rapid mechanism of action. Pending long-term data, the available safety and efficacy data of filgotinib are reassuring. Thus, filgotinib, as well as other selective JAK inhibitors, could hopefully represent an evolution towards rapid and effective oral therapies with an improved safety profile. The ever-increasing number of available drugs underlines the need for head-to-head trials that directly compare the efficacy and safety of two different molecules in order to clarify which is the best option for the patient. In this context, it is likely that therapeutic decisions will be progressively personalized and tailored based on the patient’s clinical characteristics. Soon, the combination of multiple molecules acting on different inflammatory pathways could become more common. Some case series reporting the use of combination therapies between biologics and small molecules show that this approach is feasible in the IBD field.^59,60^ On the other hand, there is no evidence regarding the association of multiple oral small molecules. More data could come from the development of national and international registries as occurred in rheumatology, especially for patients with rheumatoid arthritis, allowing for a greater understanding of the disease.^61^ A recent initiative by the European Crohn’s and Colitis Organization [ECCO] was the creation of The United Registries for Clinical Assessment and Research [UR-CARE] platform, which is an online international registry designed to collect IBD patients’ records.^62^ The development of large national and international registries including patients with similar characteristics could be essential to improve knowledge about IBD and define the best management of patients. Another relevant aspect to consider is the price of the drug along with economic issues. The median price for each patient per year after filgotinib treatment is estimated at £10 508 based on the list price.^63^ The exact cost for the annual treatment of a patient with UC is not yet known, but much will also depend on national reimbursement policies. In fact, national reimbursements will determine a different cost from country to country and could justify a country-specific positioning of filgotinib based on cost-effectiveness principles.

In conclusion, preliminary data show that filgotinib is an effective and safe drug for treatment of both biologic-naive and biologic-experienced patients with moderate to severe UC and may soon be available. Although further studies are needed for its approval by regulatory authorities, the prospect of using a highly selective drug is attractive and could usher in a new era based on individualized treatments.

## Data Availability

No new data were generated or analysed in support of this research.

## References

[1] Ungaro R , Mehandru S, Allen PB, et al. Ulcerative colitis. Lancet Lond Engl 2017;389:1756–1770. 10.1016/S0140-6736(16)32126-2 PMC6487890 27914657

[2] Danese S , Fiorino G, Peyrin-Biroulet L. Positioning therapies in ulcerative colitis. Clin Gastroenterol Hepatol Off Clin Pract J Am Gastroenterol Assoc 2020;18:1280–1290.e1. 10.1016/j.cgh.2020.01.017 31982609

[3] Singh S , George J, Boland BS, et al. Primary non-response to tumor necrosis factor antagonists is associated with inferior response to second-line biologics in patients with inflammatory bowel diseases: a systematic review and meta-analysis. J Crohns Colitis 2018;12:635–643. 29370397 10.1093/ecco-jcc/jjy004 PMC7189966

[4] D’Amico F , Peyrin-Biroulet L, Danese S, et al. New drugs in the pipeline for the treatment of inflammatory bowel diseases: what is coming? Curr Opin Pharmacol 2020;55:141–150. 33254032 10.1016/j.coph.2020.10.015

[5] D’Amico F , Parigi TL, Fiorino G, et al. Tofacitinib in the treatment of ulcerative colitis: efficacy and safety from clinical trials to real-world experience. Ther Adv Gastroenterol 2019;12:1756284819848631. 10.1177/1756284819848631 PMC6535722 31205486

[6] Danese S , D’Amico F, Bonovas S, Peyrin-Biroulet L. Positioning tofacitinib in the treatment algorithm of moderate to severe ulcerative colitis. Inflamm Bowel Dis 2018;24:2106–12. 29697791 10.1093/ibd/izy076

[7] Danese S , Argollo M, Le Berre C, Peyrin-Biroulet L. JAK selectivity for inflammatory bowel disease treatment: does it clinically matter? Gut 2019;68:1893–9. 31227590 10.1136/gutjnl-2019-318448

[8] Salas A , Hernandez-Rocha C, Duijvestein M, et al JAK-STAT pathway targeting for the treatment of inflammatory bowel disease. Nat Rev Gastroenterol Hepatol 2020;17:323–37. 32203403 10.1038/s41575-020-0273-0

[9] Sandborn WJ , Su C, Sands BE, et al; OCTAVE Induction 1, OCTAVE Induction 2, and OCTAVE Sustain Investigators. Tofacitinib as induction and maintenance therapy for ulcerative colitis. N Engl J Med 2017;376:1723–36. 28467869 10.1056/NEJMoa1606910

[10] Sands BE , Armuzzi A, Marshall JK, et al. Efficacy and safety of tofacitinib dose de-escalation and dose escalation for patients with ulcerative colitis: results from OCTAVE Open. Aliment Pharmacol Ther 2020;51:271–280. 31660640 10.1111/apt.15555 PMC9328429

[11] Sandborn WJ , Peyrin-Biroulet L, Quirk D, et al. Efficacy and safety of extended induction with tofacitinib for the treatment of ulcerative colitis. Clin Gastroenterol Hepatol 2020:S1542-3565(20)31496-8. 10.1016/j.cgh.2020.10.038 33127596

[12] Colombel J-F , Osterman MT, Thorpe AJ, et al. Maintenance of remission with tofacitinib therapy in patients with ulcerative colitis. Clin Gastroenterol Hepatol 2020:S1542-3565(20)31389-6. 10.1016/j.cgh.2020.10.004 33039585

[13] Sands BE , Taub PR, Armuzzi A, et al. Tofacitinib treatment is associated with modest and reversible increases in serum lipids in patients with ulcerative colitis. Clin Gastroenterol Hepatol Off Clin Pract J Am Gastroenterol Assoc 2020;18:123–132.e3. 10.1016/j.cgh.2019.04.059 31077827

[14] Sandborn WJ , Panés J, Sands BE, et al Venous thromboembolic events in the tofacitinib ulcerative colitis clinical development programme. Aliment Pharmacol Ther 2019;50:1068–76. 31599001 10.1111/apt.15514 PMC6899755

[15] Winthrop KL , Loftus EV, Baumgart DC, et al. Tofacitinib for the treatment of ulcerative colitis: analysis of infection rates from the ulcerative colitis clinical programme. J Crohns Colitis 2021;15:914–929. 33245746 10.1093/ecco-jcc/jjaa233 PMC8218715

[16] Traves PG , Murray B, Campigotto F, et al. JAK selectivity and the implications for clinical inhibition of pharmacodynamic cytokine signalling by filgotinib, upadacitinib, tofacitinib and baricitinib. Ann Rheum Dis 2021;80:865–875. 33741556 10.1136/annrheumdis-2020-219012 PMC8237188

[17] Czarska-thorley D . *Jyseleca. Eur Med Agency 2020*. Available at: https://www.ema.europa.eu/en/medicines/human/EPAR/jyseleca [Accessed May 9, 2021].

[18] Vermeire S , Schreiber S, Petryka R, et al Clinical remission in patients with moderate-to-severe Crohn’s disease treated with filgotinib (the FITZROY study): results from a phase 2, double-blind, randomised, placebo-controlled trial. Lancet 2017;389:266–75. 27988142 10.1016/S0140-6736(16)32537-5

[19] Dhillon S , Keam SJ. Filgotinib: first approval. Drugs 2020;80:1987–97. 33237566 10.1007/s40265-020-01439-0 PMC7858213

[20] Anderson K , Xin Y, Zheng H, et al Filgotinib, a JAK1 inhibitor, has no effect on QT interval in healthy subjects. Clin Pharmacol Drug Dev 2020;9:32–40. 31797578 10.1002/cpdd.755

[21] Namour F , Desrivot J, Van der Aa A, Harrison P, Tasset C, van’t Klooster G. Clinical confirmation that the selective JAK1 inhibitor filgotinib (GLPG0634) has a low liability for drug-drug interactions. Drug Metab Lett 2016;10:38–48. 26693854 10.2174/1872312810666151223103353

[22] Begley R , Anderson K, Watkins TR, et al Lack of drug–drug interaction between filgotinib, a selective jak1 inhibitor, and oral hormonal contraceptives levonorgestrel/ethinyl estradiol in healthy volunteers. Clin Pharmacol Drug Dev 2021;10:376–83. 32989920 10.1002/cpdd.870

[23] Anderson K , Nelson C, Gong Q, et al. Evaluation of the effect of filgotinib on the pharmacokinetics of rosuvastatin, atorvastatin, and pravastatin. Ann Rheum Dis 2021;80:1155–1156.

[24] Taylor PC , Charles-Schoeman C, Alani M, et al. Concomitant use of statins in filgotinib-treated patients with rheumatoid arthritis. Ann Rheum Dis 2021;80:572–573.

[25] Anon. Efficacy and safety of filgotinib as induction therapy for patients with moderately to severely active ulcerative colitis: results from the phase 2b/3 selection study. Journal of the Canadian Association of Gastroenterology | Oxford Academic. Available at: https://academic.oup.com/jcag/article/4/Supplement_1/18/6158632 [Accessed May 9, 2021].

[26] Peyrin-Biroulet L , Loftus EV Jr, Danese S, et al. Efficacy and safety of filgotinib as maintenance therapy for patients with moderately to severely active ulcerative colitis: results from the phase 2b/3 selection study. J Can Assoc Gastroenterol 2021;4:21–23.33644673

[27] Feagan BG , Danese S, Loftus EV Jr, et al Filgotinib as induction and maintenance therapy for ulcerative colitis (SELECTION): a phase 2b/3 double-blind, randomised, placebo-controlled trial. Lancet 2021;397(10292):2372–2384. 34090625 10.1016/S0140-6736(21)00666-8

[28] Md P , Oortwijn A, Feagan B, et al. Early achievement of partial mayo score remission and ibdq normalization in patients with ulcerative colitis treated with filgotinib in the phase 2b/3 selection study. Gastroenterology 2021;160:S–352.

[29] Inc MG . *Filgotinib provides improvements in health-related quality of life ... by Dr. William Sandborn*. Available at: https://eposters.ddw.org/ddw/2021/ddw-2021-virtual/320377/william.sandborn.filgotinib.provides.improvements.in.health-related.quality.of.html?f=listing%3D3%2Abrowseby%3D8%2Asortby%3D2%2Amedia%3D3%2Aspeaker%3D852203 [Accessed June 17, 2021].

[30] Anon. *Stefan Schreiber, DDW 2021: Phase 2b/3 SELECTION and Long Term Extension Study Results. touchIMMUNOLOGY*. Available at: https://www.touchimmunology.com/inflammatory-bowel-disease/conference-hub/stefan-schreiber-ddw-2021-phase-2b-3-selection-and-long-term-extension-study-results/ [Accessed June 17, 2021].

[31] Mease P , Coates LC, Helliwell PS, et al Efficacy and safety of filgotinib, a selective Janus kinase 1 inhibitor, in patients with active psoriatic arthritis (EQUATOR): results from a randomised, placebo-controlled, phase 2 trial. Lancet 2018;392:2367–77. 30360969 10.1016/S0140-6736(18)32483-8

[32] van der Heijde D , Baraliakos X, Gensler LS, et al. Efficacy and safety of filgotinib, a selective Janus kinase 1 inhibitor, in patients with active ankylosing spondylitis (TORTUGA): results from a randomised, placebo-controlled, phase 2 trial. Lancet Lond Engl 2018;392:2378–2387. 10.1016/S0140-6736(18)32463-2 30360970

[33] Westhovens R , Taylor PC, Alten R, et al Filgotinib (GLPG0634/GS-6034), an oral JAK1 selective inhibitor, is effective in combination with methotrexate (MTX) in patients with active rheumatoid arthritis and insufficient response to MTX: results from a randomised, dose-finding study (DARWIN 1). Ann Rheum Dis 2017;76:998–1008. 27993829 10.1136/annrheumdis-2016-210104

[34] Kavanaugh A , Kremer J, Ponce L, et al Filgotinib (GLPG0634/GS-6034), an oral selective JAK1 inhibitor, is effective as monotherapy in patients with active rheumatoid arthritis: results from a randomised, dose-finding study (DARWIN 2). Ann Rheum Dis 2017;76:1009–19. 27993828 10.1136/annrheumdis-2016-210105

[35] Anon. *Efficacy and Safety of Filgotinib for Patients with Rheumatoid Arthritis Naïve to Methotrexate Therapy: FINCH3 Primary Outcome Results. ACR Meet Abstr*. Available at: https://acrabstracts.org/abstract/efficacy-and-safety-of-filgotinib-for-patients-with-rheumatoid-arthritis-naive-to-methotrexate-therapy-finch3-primary-outcome-results/ [Accessed May 9, 2021].

[36] Anon. *THU0198 Efficacy and safety of filgotinib for patients with rheumatoid arthritis with inadequate response to methotrexate: finch 1 52-week results | Annals of the Rheumatic Diseases*. Available at: https://ard.bmj.com/content/79/Suppl_1/320 [Accessed May 9, 2021].

[37] Genovese MC , Kalunian K, Gottenberg JE, et al Effect of filgotinib vs placebo on clinical response in patients with moderate to severe rheumatoid arthritis refractory to disease-modifying antirheumatic drug therapy: the FINCH 2 randomized clinical trial. JAMA 2019;322:315–25. 31334793 10.1001/jama.2019.9055 PMC6652745

[38] Kavanaugh A , Westhovens RR, Winthrop KL, et al. Safety and efficacy of filgotinib: up to 4-year results from an open-label extension study of phase II Rheumatoid Arthritis Programs. J Rheumatol 2021;48(8):1230–1238. 33526618 10.3899/jrheum.201183

[39] Anon. *Gilead Receives Complete Response Letter for Filgotinib for the Treatment of Moderately to Severely Active Rheumatoid Arthritis*. Available at: https://www.gilead.com/news-and-press/press-room/press-releases/2020/8/gilead-receives-complete-response-letter-for-filgotinib-for-the-treatment-of-moderately-to-severely-active-rheumatoid-arthritis [Accessed June 15, 2021].

[40] Nv G . Galapagos reports primary endpoint for the ongoing filgotinib manta and manta-ray safety studies. GlobeNewswire News Room 2021. Available at: https://www.globenewswire.com/news-release/2021/03/04/2186756/0/en/GALAPAGOS-REPORTS-PRIMARY-ENDPOINT-FOR-THE-ONGOING-FILGOTINIB-MANTA-AND-MANTA-RAy-SAFETY-STUDIES.html [Accessed June 15, 2021].

[41] Smolen JS , Landewé RBM, Bijlsma JWJ, et al. EULAR recommendations for the management of rheumatoid arthritis with synthetic and biological disease-modifying antirheumatic drugs: 2019 update. Ann Rheum Dis 2020;79:685–699. 31969328 10.1136/annrheumdis-2019-216655

[42] Chateau T , Bonovas S, Le Berre C, Mathieu N, Danese S, Peyrin-Biroulet L. Vedolizumab treatment in extra-intestinal manifestations in inflammatory bowel disease: a systematic review. J Crohns Colitis 2019;13:1569–77. 31076751 10.1093/ecco-jcc/jjz095

[43] Gisbert JP , Chaparro M. Safety of new biologics (vedolizumab and ustekinumab) and small molecules (tofacitinib) during pregnancy: a review. Drugs 2020;80:1085–100. 32562207 10.1007/s40265-020-01346-4

[44] Mahadevan U , Long MD, Kane SV, et al. Pregnancy and neonatal outcomes after fetal exposure to biologics and thiopurines among women with inflammatory bowel disease. Gastroenterology 2021;160:1131–1139. 33227283 10.1053/j.gastro.2020.11.038 PMC7956164

[45] Wils P , Seksik P, Stefanescu C, et al; PREGNANCY-GETAID study group. Safety of ustekinumab or vedolizumab in pregnant inflammatory bowel disease patients: a multicentre cohort study. Aliment Pharmacol Ther 2021;53:460–70. 33345331 10.1111/apt.16192

[46] Overton PM , Shalet N, Somers F, et al. Patient preferences for subcutaneous versus intravenous administration of treatment for chronic immune system disorders: a systematic review. Patient Prefer Adherence 2021;15:811–834. 33907384 10.2147/PPA.S303279 PMC8064718

[47] Bell CF , Lau M, Lee M, et al. Insights into the choice between intravenous infusion and subcutaneous injection: physician and patient characteristics driving treatment in SLE. Clin Rheumatol 2021;40:581–590. 32623647 10.1007/s10067-020-05226-w PMC7817604

[48] Chupin A , Perduca V, Meyer A, Bellanger C, Carbonnel F, Dong C. Systematic review with meta-analysis: comparative risk of lymphoma with anti-tumour necrosis factor agents and/or thiopurines in patients with inflammatory bowel disease. Aliment Pharmacol Ther 2020;52:1289–97. 32840893 10.1111/apt.16050

[49] Khan N , Patel D, Trivedi C, et al Incidence of acute myeloid leukemia and myelodysplastic syndrome in patients with inflammatory bowel disease and the impact of thiopurines on their risk. Am J Gastroenterol 2021;116:741–7. 33982944 10.14309/ajg.0000000000001058

[50] Zheng KYC , Guo CG, Wong IOL, et al Risk of malignancies in patients with inflammatory bowel disease who used thiopurines as compared with other indications: a territory-wide study. Therap Adv Gastroenterol 2020;13:1756284820967275. 10.1177/1756284820967275 PMC7682226 33281936

[51] Ho Lee Y , Gyu Song G. Comparative efficacy and safety of tofacitinib, baricitinib, upadacitinib, filgotinib and peficitinib as monotherapy for active rheumatoid arthritis. J Clin Pharm Ther 2020;45:674–81. 32495356 10.1111/jcpt.13142

[52] Sung Y-K , Lee YH. Comparative study of the efficacy and safety of tofacitinib, baricitinib, upadacitinib, and filgotinib versus methotrexate for disease-modifying antirheumatic drug-naïve patients with rheumatoid arthritis. Z Rheumatol 2020. 10.1007/s00393-020-00889-x 32970188

[53] Lee YH , Song GG. Relative efficacy and safety of tofacitinib, baricitinib, upadacitinib, and filgotinib in comparison to adalimumab in patients with active rheumatoid arthritis. Z Rheumatol 2020;79:785–796. 32055928 10.1007/s00393-020-00750-1

[54] Lee YH , Song GG. Comparative efficacy and safety of tofacitinib, baricitinib, upadacitinib, and filgotinib in active rheumatoid arthritis refractory to biologic disease-modifying antirheumatic drugs. Z Rheumatol 2021;80:379–92. 32367211 10.1007/s00393-020-00796-1

[55] Olivera PA , Lasa JS, Bonovas S, Danese S, Peyrin-Biroulet L. Safety of janus kinase inhibitors in patients with inflammatory bowel diseases or other immune-mediated diseases: a systematic review and meta-analysis. Gastroenterology 2020;158:1554–1573.e12. 31926171 10.1053/j.gastro.2020.01.001

[56] Agrawal M , Kim ES, Colombel JF. JAK inhibitors safety in ulcerative colitis: practical implications. J Crohns Colitis 2020;14:755–60. 10.1093/ecco-jcc/jjaa017 PMC7395307 32006031

[57] Guillo L , Rabaud C, Choy EH, et al. Herpes zoster and vaccination strategies in inflammatory bowel diseases: a practical guide. Clin Gastroenterol Hepatol 2020:S1542-3565(20)31440-3. 10.1016/j.cgh.2020.10.027 33080353

[58] FDA. *Initial safety trial results find increased risk of serious heart-related problems and cancer with arthritis and ulcerative colitis medicine Xeljanz, Xeljanz XR (tofacitinib). FDA 2021*. Available at: https://www.fda.gov/drugs/drug-safety-and-availability/initial-safety-trial-results-find-increased-risk-serious-heart-related-problems-and-cancer-arthritis [Accessed May 9, 2021].

[59] Yang E , Panaccione N, Whitmire N, et al Efficacy and safety of simultaneous treatment with two biologic medications in refractory Crohn’s disease. Aliment Pharmacol Ther 2020;51:1031–8. 32329532 10.1111/apt.15719 PMC8032452

[60] Glassner K , Oglat A, Duran A, et al The use of combination biological or small molecule therapy in inflammatory bowel disease: A retrospective cohort study. J Dig Dis 2020;21:264–71. 32324969 10.1111/1751-2980.12867

[61] Nash P , Kerschbaumer A, Dörner T, et al Points to consider for the treatment of immune-mediated inflammatory diseases with Janus kinase inhibitors: a consensus statement. Ann Rheum Dis 2021;80:71–87. 33158881 10.1136/annrheumdis-2020-218398 PMC7788060

[62] Anon. *UR-CARE*. Available at: https://www.ecco-ibd.eu/science/ur-care.html [Accessed May 27, 2021].

[63] Anon. *Information about filgotinib | Filgotinib for treating moderate to severe rheumatoid arthritis | Guidance | NICE*. Available at: https://www.nice.org.uk/guidance/ta676/chapter/2-Information-about-filgotinib [Accessed May 27, 2021]. 40737465

